# Acute ST-elevation myocardial infarction (STEMI) in a young woman with unknown mitral stenosis and atrial fibrillation: Case report

**DOI:** 10.1016/j.amsu.2022.103465

**Published:** 2022-03-03

**Authors:** Raid Faraj, Ahmed Djibril, Fatima-Azzahra Benmessaoud, Mohamed Benasser, Jamila Zarzur, Latifa Oukerraj, Rachida Amri, Mohamed Cherti

**Affiliations:** Mohammed V University, Rabat, Morocco

**Keywords:** Mitral stenosis, Embolism, MINOCA, Acute myocardial infarction

## Abstract

**Introduction and importance:**

Chronic rheumatic heart disease is the most common cause of mitral stenosis. It remains a major public health problem. In almost half of the cases, paroxysmal or chronic atrial fibrillation occurs during the evolution of mitral stenosis, thereby exposing to an increased risk of thrombo-embolic events.

Whereas the most frequent site for embolism is the cerebral circulation, any organ may be involved, especially the coronary circulation, resulting in a myocardial infarction (MI).

**Case presentation:**

Here, we report a rare case of a 50-year-old patient, with no risk factors for cardiovascular disease, presenting an acute ST-elevation myocardial infarction (STEMI) as initial presentation of unknown mitral stenosis with atrial fibrillation and strongly suggesting an embolic origin. The diagnosis was made based on the national cerebral and cardiovascular center (NCVC) criteria for the clinical diagnosis of coronary artery embolism (CE). Coronary angiography showed a distal thrombus in the right coronary artery that has been medically treated. The outcome was favorable and the patient was referred after that for mitral valve replacement.

## Highlights

• Coronary embolism (CE) is an uncommon and rare cause of acute coronary syndrome (ACS).
• Atrial fibrillation represents the most common cause of CE, followed by cardiomyopathy and valvular heart disease.
• Long term outcomes of STEMI patients related to CE are worst compared to STEMI patients non related to CE.

## 1 Introduction

Coronary embolism (CE) is an uncommon and rare cause of acute coronary syndrome (ACS). It often attains the microcirculation, even though angiographically visible embolization of epicardial coronary artery branches may occur. The prevalence of CE among other causes of ACS is estimated to be around 3% [1]. Atrial fibrillation represents the most common cause of CE, followed by cardiomyopathy and valvular heart disease [1]. It should be evoked in patients with myocardial infarction with no obstructive coronary atherosclerosis (MINOCA) and at least one or more conditions associated with high risk of systemic embolism [2].

Due to the high prevalence of mitral stenosis and atrial fibrillation, cardiologists and emergency physicians should consider the diagnosis of CE when this patient population has acute coronary syndrome's (ACS) presentation.

Our case report was written according to CARE guidelines [3].

## 2 Case presentation

A 50 year old woman, presented to Ibn Sina University Hospital with sudden onset of severe retrosternal chest pain, that appeared 3 days before her admission. She did not report any history of diabetes mellitus, hypertension, smoking, dyslipidemia or genetic predisposition to coronary artery disease.

**Fig. 1:**
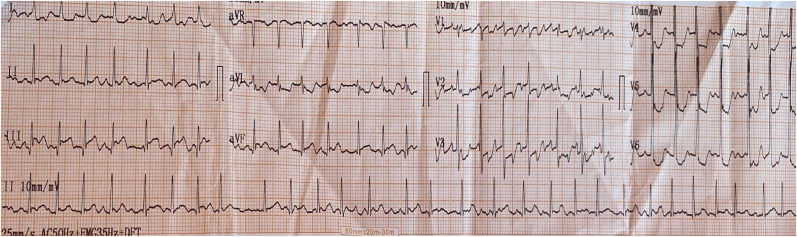
ECG showing atrial fibrillation atrial fibrillation with a fast ventricular rate with ST segment elevation in inferior leads associated with mirror reflection of ST-elevation in anterior and lateral leads.

**Fig. 2:**
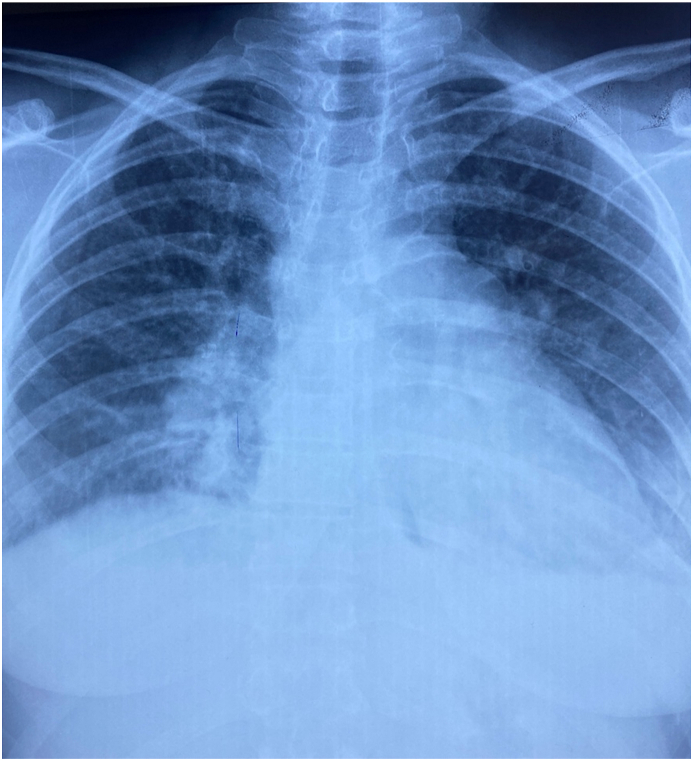
Chest x-ray showing mildly enlarged cardiomegaly with signs of pulmonary artery hypertension and biatrial enlargement.

**Fig. 3:**
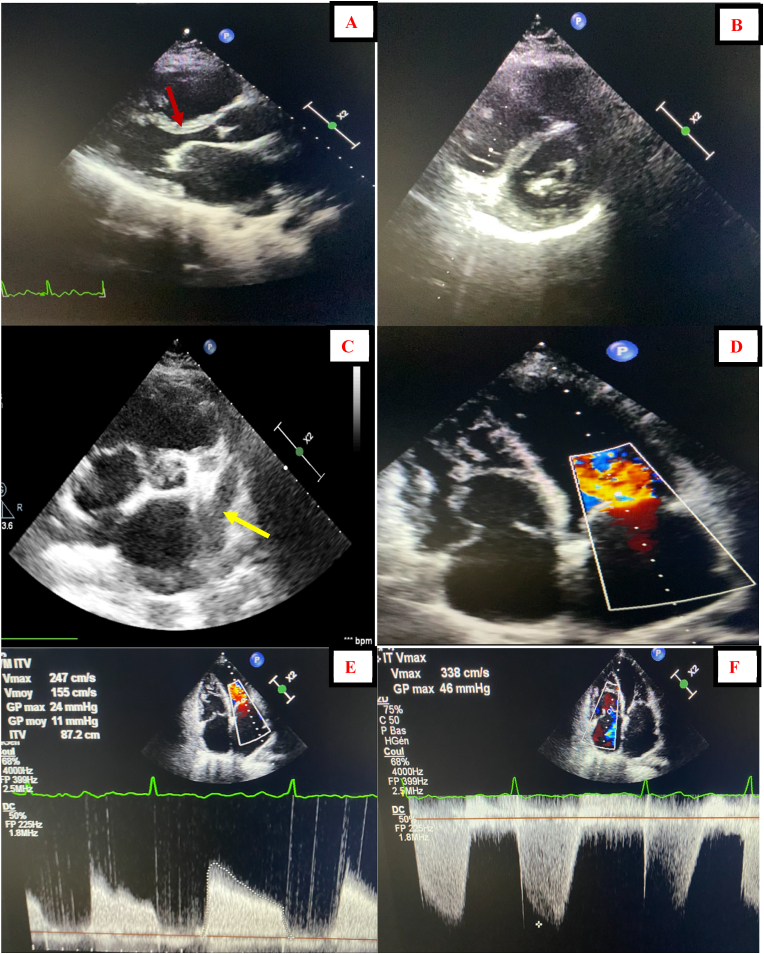
TEE findings: (A) parasternal long axis view showing a “dog leg deformity” of the anterior mitral leaflet (red arrow) with rheumatic changes of the mitral valve (B) parasternal short axis view showing a mitral valve area stenosis. (C) parasternal short axis view showing the presence of a sludge in the left atrial appendage (yellow arrow). (D) Apical four chamber view with color flow Doppler showing a high velocity narrow “jet” across a stenotic mitral valve with biatrial and right ventricle enlargement. (E) Continuous wave Doppler showing a mean gradient across the mitral valve estimated at 11 mmhg. (F) Continuous wave Doppler across the tricuspid valve estimating an important PASP. (For interpretation of the references to color in this figure legend, the reader is referred to the Web version of this article.)

The patient was conscious. She was no longer suffering from chest pain. Her blood pressure was 110/70 mm Hg, her heart rate was irregularly irregular at 130 b/m, oxygen saturation value 98% on room air and a temperature of 37.3 °C. A grade III diastolic rumbling murmur was audible at the apex associated to a pulmonary component of second heart sound. The rest of physical exam was normal. The electrocardiogram (ECG) showed atrial fibrillation with a fast ventricular rate with ST-segment elevation in inferior leads II, III and Avf associated with mirror reflection of ST-elevation in anterior and lateral leads (Fig. 1). Chest X-ray found a mildly enlarged cardiomegaly with signs of pulmonary artery hypertension and biatrial enlargement (Fig. 2). High sensitivity cardiac troponin T (hs-cTnT) on admission was, as expected, very high (>50.000 ng/ml) with a normal threshold value below 0.017ng/ml. Signs of inflammatory response were present, white blood cells counted to 13,500/mm3 in addition to C reactive protein at 180 mg/l. A transthoracic echocardiogram (TTE) objectivated severe mitral stenosis with a mean gradient across the mitral valve estimated at 11 mmhg and a mitral valve area of 1 cm2. The two atria were dilated with the presence of a sludge in the left atrial appendage. The right ventricle was also dilated and the pulmonary artery systolic pressure (PASP) was estimated at 51 mmHg. We also noted akinesia of the inferior wall and hypokinesia of the inferoseptal wall with a mild left ventricular dysfunction (Fig. 3). A transesophageal echocardiogram (TEE) was indicated but refused by the patient.

**Fig. 4:**
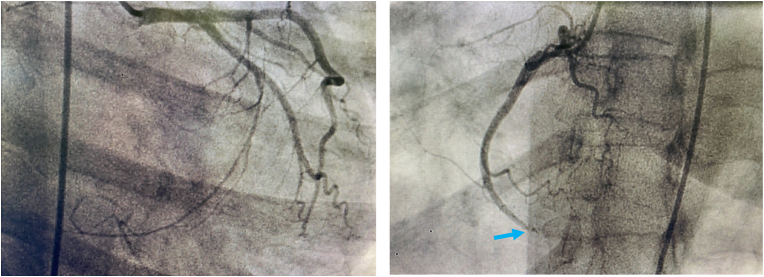
Coronary angiogram findings: (A) Normal left coronary artery without atherosclerotic plaques (B) Presence of a thrombus in the distal right coronary artery.

Coronary angiography was performed. It revealed a thrombus in the distal right coronary artery. The left and right coronary systems were clear of atherosclerotic plaques (Fig. 4). The diagnosis of coronary artery embolism (CE) was made, based on the proposed national cerebral and cardiovascular center (NCVC) criteria for the clinical diagnosis of coronary artery embolism (CE).

She was treated with dual antiplatelet (DAPT), beta-blocker and enoxaparin was administrated in overlap with vitamin K antagonists (VKA) until reaching the therapeutic range of the international normalized ratio (INR). After evaluation of the ischemic and hemorrhagic risk, triple therapy was maintained for one week, followed by a bitherapy including VKA and Clopidogrel. The tolerability was good and no adverse events were reported. The patient was satisfied after the improvement of her clinical condition. She was advised on the necessity of anticoagulation and prophylactic measures against infectious endocarditis. She was referred after that for mitral valve replacement.

## 3 Discussion

Myocardial infarction with no obstructive coronary atherosclerosis (MINOCA) is a distinct clinical syndrome characterized by evidence of myocardial infarction with normal or near normal coronary arteries on coronarography in the absence of obvious noncoronary causes of MI like a severe hemorrhage or severe respiratory failure [4]. It may involve the coronary circulation and or epicardial vessels.

Patients with atrial fibrillation and/or valvular heart disease have an increased risk of thombus formation in the left atrial appendage with a major risk of embolization. In case of CE, the coronary arteries are obviously not normal due to an abrupt vessel stump or thrombotic material inside the epicardial coronary artery [5].

CE can be manifested as a STEMI like in our case or an NSTEMI as seen in other case reports. Indeed, in a recent article published in the American journal of case reports, authors have reported the case of a 22 year-old woman, followed for mitral stenosis with atrial fibrillation, who presented a NSTEMI related to total occlusion mid left anterior descending artery (LAD). Yet only few articles have reported, as in our case, STEMI as the primary presentation of unknown mitral stenosis with atrial fibrillation. Furthermore, the left coronary artery is concerned in 75% cases of CE, as opposed to our case in which it's rather the right coronary artery that was incriminated [6].

ACS related to CE remains a very challenging diagnosis for cardiologists. This can be explained by its rarity and the absence of consensus regarding diagnosis and treatment. Accordingly, Shibata et al. proposed the national cerebral and cardiovascular center (NCVC) criteria for the clinical diagnosis of coronary artery embolism (CE). It contains major and minor criteria, allowing to classify the diagnosis of CE into probable or definite CE [1]. In our case, we gathered one major criteria plus two minor criteria, permitting to conclude to a definite CE.

Several treatment options exist. It includes medical management (glycoprotein IIb/IIIa inhibitors, heparin and thrombolytic agents) and/or invasive management (thrombus aspiration and angioplasty) [7]. Our patient was seen 3 days after the onset of chest pain with a distal located thrombus and a preserved anterograde flow. Therefore, a conservative approach was adopted including anticoagulation with DAPT. Etiological treatment of CE is a cornerstone of management. Thus, Anticoagulation and rate control strategy was initiated for the treatment of AF and a mitral valve replacement scheduled to avoid CE recurrences.

Long term outcomes of STEMI patients related to CE are worst than STEMI patients without CE. In a retrospective observational cohort study, published in Circulation journal, The 5-year rate CE recurrence was 8.7% and the 5-year rate of MACCE was 27.1%. Furthermore, the 5-year rates of all-cause death and cardiac death in the CE group were high compared with non-CE group [8]. It's important to highlight that CE recurrence is inextricably linked to AF patients with inadequate international normalized ratio (INR).

Accordingly, establishing an etiological diagnosis of ACS remains crucial.

## 4 Conclusion

Our case report demonstrates that ACS related to CE may be the initial presentation of unknown mitral stenosis. Therefore, cardiologists, must evoke this diagnosis in patients with MINOCA and at least one or more conditions associated with high risk of systemic embolism.

## Ethical approval

N/a.

## Sources of funding

None.

## Author contribution

Raid Faraj: Study concept, Data collection, Data analysis, Literature research, Writing the paper. Ahmed Djibril: Data collection, Data analysis. Fatima-Azzahra Benmessaoud: Data collection, Data analysis. Mohamed Benasser: Data collection, Data analysis. Jamila Zarzur: Supervision and data validation. Latifa Oukerraj: Supervision and data validation. Rachida Amri: Supervision and data validation. Mohamed Cherti: Supervision and data validation.

## Consent

Written informed consent was obtained from the patient for publication of this case report and accompanying images. A copy of the written consent is available for review by the Editor-in-Chief of this journal on request.

## Registration of research studies

This is not an original research project involving human participants in an interventional or an observational study but a case report. This registration is was not required.

## Guarantor

Raïd Faraj.

## Provenance and peer review

Not commissioned, externally peer-reviewed.

## Declaration of competing interest

None.

## References

[1] Shibata T. (2015). Prevalence, clinical features, and prognosis of acute myocardial infarction attributable to coronary artery embolism. Circulation.

[2] Myocardial infarction with no obstructive coronary atherosclerosis - UpToDate.” https://www.uptodate.com/contents/myocardial-infarction-with-no-obstructive-coronary-atherosclerosis?search=minoca&source=search_result&selectedTitle=1∼2&usage_type=default&display_rank=1#H11320645 (accessed Feb. 26, 2022).

[3] Ds R. (Sep. 2017). CARE guidelines for case reports: explanation and elaboration document. J. Clin. Epidemiol..

[4] Niccoli G., Scalone G., Crea F. (Feb. 2015). Acute myocardial infarction with no obstructive coronary atherosclerosis: mechanisms and management. Eur. Heart J..

[5] Tamis-Holland J.E. (Apr. 2019). Contemporary diagnosis and management of patients with myocardial infarction in the absence of obstructive coronary artery disease: a scientific statement from the American heart association. Circulation.

[6] Prizel K.R., Hutchins G.M., Bulkley B.H. (1978). Coronary artery embolism and myocardial infarction. Ann. Intern. Med..

[7] Liang M., Kelly D., Puri A., Devlin G. (Nov. 2011). Mitral stenosis as a risk factor for embolic myocardial infarction--anticoagulation for some patients, individual treatment for all. Heart Lung Circ..

[8] Shibata T. (2015). Prevalence, clinical features, and prognosis of acute myocardial infarction attributable to coronary artery embolism. Circulation.

